# Fulminant Idiopathic Intracranial Hypertension Presenting With Bitemporal Hemianopia Mimicking Chiasmal Pathology in Early Pregnancy: A Case Report

**DOI:** 10.7759/cureus.111095

**Published:** 2026-06-18

**Authors:** Tharindu Ruwanpathiranage, Benedict S Sebastiampillai, Taha Elsahy

**Affiliations:** 1 Acute Medicine, Peterborough City Hospital, Peterborough, GBR; 2 Acute Medicine, North West Anglia NHS Foundation Trust, Peterborough, GBR

**Keywords:** bitemporal hemianopia, early pregnancy, fulminant idiopathic intracranial hypertension, idiopathic intracranial hypertension (iih), papilledema

## Abstract

Idiopathic intracranial hypertension (IIH) is characterized by elevated intracranial pressure without an identifiable cause and typically presents with headache, papilledema, and transient visual obscurations (TVOs). Fulminant IIH is a rare, rapidly progressive subtype that can lead to permanent visual loss. Although visual field defects are common in IIH, bitemporal hemianopia is an uncommon and diagnostically challenging presentation that may initially suggest chiasmal compression.

A 29-year-old woman with no significant medical history presented with two weeks of progressive peripheral visual loss, headache, dizziness, and gait imbalance. Examination revealed bitemporal hemianopia and severe papilledema. She was subsequently found to be in the very early stages of pregnancy. Urgent neuroimaging revealed radiological signs of increased intracranial pressure, without evidence of a chiasmal or sellar mass. Lumbar puncture confirmed markedly elevated opening pressure (>40 cmH₂O) with normal cerebrospinal fluid (CSF) composition. She underwent serial CSF drainage, received acetazolamide, and subsequently required ventriculoperitoneal shunt insertion, resulting in significant improvement in visual and gait symptoms.

This case demonstrates an atypical presentation of fulminant IIH, where bitemporal hemianopia, which is typically associated with chiasmal lesions, occurred secondary to severe papilledema rather than structural compression. Although rare in IIH, such visual field patterns necessitate prompt differentiation from chiasmal pathology. Rapid diagnosis and early intervention are crucial for preventing irreversible visual loss in fulminant IIH. The patient's pregnancy raises the possibility that it may have precipitated the development of fulminant IIH.

## Introduction

Idiopathic intracranial hypertension (IIH) is a disorder characterized by increased pressure inside the skull without any identifiable underlying cause. It most commonly occurs in overweight women of reproductive age. Fulminant IIH refers to an aggressive and rapidly worsening form of the condition [1,2].

Common symptoms include headaches, loss of vision, transient visual obscurations (TVOs), diplopia, divergence insufficiency, and pulsatile tinnitus [3]. Atypical presentations of IIH have been previously described and may pose significant diagnostic challenges [4]. These atypical features can be a challenge when diagnosing IIH and can attribute the features to a different condition. An IIH patient presenting with a bitemporal hemianopia type of visual field defect is uncommon.

This patient presented with peripheral visual loss, headache, dizziness, and imbalance of two weeks' duration. Examination findings of bitemporal hemianopia along with headache were initially suggestive of a chiasmal lesion. Therefore, urgent brain imaging and ophthalmology review were done to exclude a chiasmal lesion. However, ophthalmology findings revealed severe papilledema, and the magnetic resonance imaging (MRI) of the brain showed changes suggestive of raised intracranial pressure. Radiological evidence of papilledema, along with an unremarkable appearance of the sella and the pituitary with no evidence of related mass, warranted urgent lumbar puncture to diagnose fulminant IIH in this patient. She was also incidentally found to be approximately three weeks of gestational age at the time of diagnosis, raising the possibility that the pregnancy contributed to the development of fulminant IIH in this patient.

This case was previously presented as a poster at the stroke and neurology conference for the generalist by the Royal College of Physicians and Surgeons of Glasgow on 27 February 2026.

## Case presentation

A 29-year-old female patient with insignificant past medical history presented with a new onset of peripheral visual loss, dizziness, difficulty in walking, and imbalance for two weeks. She needed support with walking due to these clinical features.

She also had a headache and visual blurring during this period. Her headache was gradual in onset, mainly occipital and radiating bitemporally. It was exacerbated by bending forward, lying down, and straining. She experienced transient visual obscuration, which lasted for a few seconds, and her peripheral visual loss was deteriorating progressively. She had occasional pulsatile tinnitus. She did not have facial, arm or leg weakness or speech or swallowing difficulty. She did not have a fever or any systemic symptoms of an infection. Her hearing was normal, and she denied any antecedent history of headaches. She was fit and well before this presentation. She was a non-smoker and non-alcoholic, and the rest of her history was unremarkable. She was also found to be pregnant on urine human chorionic gonadotropin (hCG) testing. At the time of diagnosis, she was gravida 3, para 2 (G3P2), and approximately three weeks of gestational age. Both previous pregnancies had resulted in uncomplicated vaginal deliveries, with no history of similar symptoms during either pregnancy. She reported regular menstrual cycles and was not using any form of contraception.

On examination, her body mass index was 33 kg/m². Her blood pressure was normal with no evidence of postural hypotension. Her visual fields on confrontation revealed bitemporal hemianopia with papilledema. Her pupils were normal in size and reactive. She did not have a relative afferent pupillary defect. Her extraocular muscle movements were normal. She had positive Romberg’s signs with gait ataxia, and the rest of the cerebellar signs were negative. She did not have any other focal neurological signs in her cranial nerve or limb examinations. Her vision deteriorated rapidly over the next few days.

Investigations

**Figure 1:**
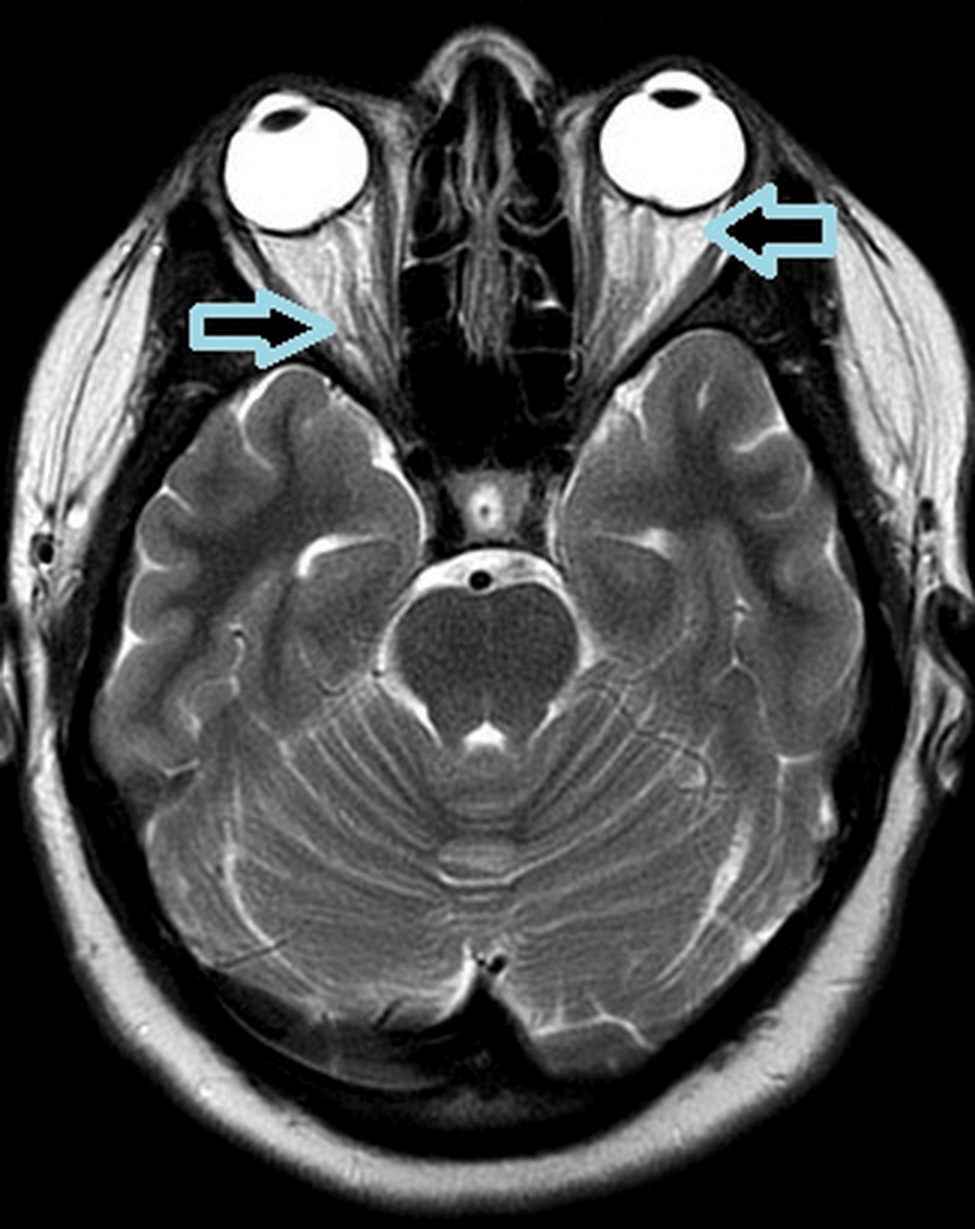
Distension of the bilateral optic nerve sheaths on MRI brain (blue arrows)

There was no significant abnormality in her initial blood results (Table 1). She underwent an urgent non-contrast computed tomography (CT) scan of the brain, and it was found to be normal. In view of excluding a chiasmal pathology, an urgent MRI scan of the brain was requested along with an ophthalmology referral. The MRI of the brain showed distension of the bilateral optic nerve sheaths (Figure 1) and prominent bilateral papilledema/optic nerves and protrusion, which suggested raised intracranial pressure. There was an unremarkable appearance of the sella and the pituitary with no evidence of a related mass. Ophthalmology findings revealed bilateral Frisen grade 5 papilledema with a retinal nerve thickness of 400 microns in the left eye and 320 microns in the right eye. Her CT venogram did not show any evidence of venous thrombosis.

**Table 1:** Initial blood test results

Blood Parameter	Result	Reference Range
Hemoglobin (g/L)	114	130–180 (g/L)
White cell count (10⁹/L)	9.7	4–11(10⁹/L)
Platelet count (10⁹/L)	306	150–400 (10⁹/L)
Sodium (mmol/L)	136	133–146 (mmol/L)
Creatinine (µmol/L)	60	59–104 (µmol/L)
C-reactive protein (mg/L)	17	<5 (mg/L)
Amylase (IU/L)	56	<100 (IU/L)
Total bilirubin (µmol/L)	09	<21 (µmol/L)
Alkaline phosphatase (IU/L)	66	30–130 (IU/L)
Alanine transferase (U/L)	10	<33 (U/L)
Albumin (g/L)	41	35–50 (g/L)

An urgent lumbar puncture was arranged for her, and it had an opening pressure of more than 40 cmH₂O with normal CSF examination findings (Table 2). A routine dating transabdominal ultrasound scan performed at 12 weeks of gestation suggested that the patient had been approximately three weeks of gestational age at the time of her initial presentation.

**Table 2:** Cerebrospinal fluid (CSF) results

Parameter	Result	Reference Range
CSF protein (g/L)	0.21	0.15-0.45 (g/L)
CSF glucose (mmol/L)	3.5	2.2-4.0 (mmol/L)
Appearance	Clear, colorless	-
White blood cells (cells x 10^6^/L)	4	0 to 5 cells × 10⁶/L
Red blood cells (cells x 10^6^/L)	64	0 cells × 10⁶/L
Organisms	Bacteria not seen	-
CSF culture	No growth	-

Treatment

CSF drainage was performed daily for two consecutive days, with 15 mL and 25 mL drained during the respective procedures. In addition, she was started on acetazolamide 750 mg twice daily as per the neurology team's recommendation to reduce the progressive visual impairment. Subsequently, she underwent urgent neurosurgical intervention on the third day following diagnosis, where a permanent ventriculoperitoneal (VP) shunt was inserted under general anesthesia.

Outcome and follow-up

Visual symptoms and gait disturbance improved significantly within one to two weeks following VP shunt insertion. At the three-month follow-up, she reported complete resolution of symptoms with no residual neurological deficits. Ophthalmological review, including optical coherence tomography (OCT), demonstrated resolution of optic disc swelling, and fundus examination was normal. She continued routine antenatal follow-up under the obstetrics team, and her pregnancy progressed uneventfully. As she remained asymptomatic throughout the remainder of the pregnancy and the VP shunt functioned satisfactorily, there was no neurosurgical contraindication to vaginal delivery. However, due to breech presentation, she underwent an elective caesarean section at 39 weeks and four days of gestation and delivered a healthy male infant. Given her stable clinical course and absence of neurological symptoms, no additional specialist input was required during the remainder of the pregnancy or in the postpartum period.

## Discussion

IIH is defined as raised intracranial pressure without an identifiable secondary cause. The main clinical features include headache, pulsatile tinnitus, and transient visual obscurations [2,5-7]. Fulminant IIH is a subtype of IIH that occurs in approximately 2%-3% of patients. It is characterized by severe and rapidly progressive vision loss developing within one month of the onset of IIH symptoms [2]. This patient’s vision rapidly deteriorated within three weeks.

One of the key features of this case is that the presentation of this patient was predominantly in favor of a chiasmal pathology initially, as she had clinical features such as peripheral visual loss, headache, and bitemporal hemianopia. Although typical IIH features like headache, TVOs, and tinnitus were also noted, the working diagnosis with the initial presentation was to exclude a compressive lesion over the optic chiasma. Urgent neuroimaging and ophthalmology referrals were done accordingly.

Ophthalmic symptoms, particularly papilledema and transient visual obscurations along with pulsatile noises in the head or ears, are hallmark features of IIH, also known as pseudotumor cerebri [8]. Papilledema is swelling of the optic disc caused by increased intracranial pressure. Most patients with severe papilledema experience visual dysfunction, including reduced color vision, visual field defects, and impaired contrast sensitivity. Visual acuity is often affected later in the course of the disease [9]. A study evaluating visual field abnormalities in patients with IIH reported enlarged blind spots in 14 patients (23.33%), unilateral scotoma in two patients (3.33%), bilateral scotoma in 34 patients (56.67%), bitemporal hemianopia in two patients (3.33%), and peripheral visual field constriction in eight patients (13.33%) [10]. Therefore, bitemporal hemianopia is a very uncommon visual field abnormality in patients with IIH.

Bitemporal hemianopia is a well-recognized visual field defect with a broad differential diagnosis. Common causes include chiasmal lesions such as pituitary macroadenoma, craniopharyngioma, Rathke’s cleft cyst, meningioma, and chiasmal glioma. Less common causes include demyelinating disease, inflammatory conditions such as sarcoidosis, lupus, and lymphocytic hypophysitis, as well as ethambutol toxicity and aneurysms [11]. It is commonly associated with chiasmal disorders. This patient was initially detected to have bitemporal hemianopia on clinical evaluation.

In fulminant IIH, bitemporal visual field defects can arise as a consequence of severe papilledema rather than direct compression of the optic chiasm. Elevated intracranial pressure transmitted through the optic nerve sheath leads to axoplasmic flow stasis at the optic disc, resulting in axonal swelling. Papilledema produces multiple effects on the visual pathway, the most significant being dysfunction of swollen nerve fibers, followed by progressive loss of retinal nerve fibers and eventual optic atrophy. Both large and small fibers within the optic disc are involved; however, peripheral retinal nerve fibers tend to be affected earlier and more extensively than central fibers [12].

It is very important to consider fulminant IIH as a differential diagnosis in a patient presenting with bitemporal hemianopia with rapid vision deterioration, showing no compressive lesion on neuroimaging. Urgent lumbar puncture to measure and drain the CSF is the key, as fulminant IIH patients are at a high risk for permanent vision loss and require prompt medical diagnosis and treatment. This case highlights the value of urgent brain imaging, ophthalmology review, and lumbar puncture to exclude fulminant IIH in patients presenting with atypical features of IIH with the aim of stopping the progression of irreversible visual damage. Early diagnosis and timely intervention led to the complete resolution of neurological symptoms and an uncomplicated progression of pregnancy to term. She was managed predominantly by the obstetric team throughout pregnancy, with routine neurosurgical and ophthalmology follow-up. She did not require additional specialist input beyond obstetrics, neurosurgery, and ophthalmology, and no specific postpartum contraceptive counseling was required as she remained asymptomatic.

A study by Thaller et al. found that a diagnosis of IIH during pregnancy was rare but associated with more severe papilledema [13]. Evidence suggests that the pathophysiology of IIH during pregnancy is multifactorial, with obesity, metabolic dysregulation, hormonal fluctuations, and coagulation abnormalities potentially increasing the risk of developing the condition [14,15]. Although she was pregnant as early as approximately three weeks of gestation at the time of diagnosis, it remains possible that the pregnancy acted as a precipitating factor for her fulminant IIH.

## Conclusions

Fulminant IIH can present with atypical visual field abnormalities such as bitemporal hemianopia, mimicking chiasmal pathology. Early recognition through neuroimaging, ophthalmology review, and lumbar puncture is essential. Timely medical and surgical intervention can prevent permanent visual impairment and restore functional independence. Very early pregnancy may have acted as a contributing or precipitating factor in the development of fulminant IIH in this patient.
